# The Role of Nrf2 in the PM-Induced Vascular Injury Under Real Ambient Particulate Matter Exposure in C57/B6 Mice

**DOI:** 10.3389/fphar.2021.618023

**Published:** 2021-02-26

**Authors:** Mengyu Gao, Yuanyuan Ma, Jing Luo, Daochuan Li, Menghui Jiang, Qixiao Jiang, Jingbo Pi, Rui Chen, Wen Chen, Rong Zhang, Yuxin Zheng, Lianhua Cui

**Affiliations:** ^1^ Department of Toxicology, School of Public Health, Qingdao University, Qingdao, China; ^2^ Department of Toxicology, School of Public Health, Sun Yat-sen University, Guangzhou, China; ^3^ School of Public Health, China Medical University, Shenyang, China; ^4^ Department of Toxicology, School of Public Health, Capital Medical University, Beijing, China; ^5^ Department of Toxicology, School of Public Health, Hebei Medical University, Shijiazhuang, China

**Keywords:** Pm, AngII, Nrf2, endothelia cell dysfunction, oxidative stress

## Abstract

Short-and long-term exposure to particulate matter (PM) has been associated with cardiovascular disease (CVD). It is well recognized that oxidative stress is a potential major mechanism in PM-induced vascular injuries, in which the nuclear factor E2-related factor 2 (Nrf2) signaling pathway plays a critical role. In the current study, a Nrf2 knockout mouse model was used in combination with an individual ventilated cage (IVC)-based real-ambient PM exposure system to assess the potential vascular injury and the potential role of Nrf2 in the angiotensin II (Ang II)-associated vascular injury. After 6-or 11-week exposure to PM, the histopathology assay revealed that PM exposure resulted in the thickening of the walls of vascular. After 6 weeks exposure to PM, the ELISA assay revealed that PM exposure resulted in the elevated plasma concentration of Ang II. The expression levels of genes of interest were then further investigated with quantitative real-time PCR. Notably, the results showed that Angiotensinogen (AGT), Angiotensin converting enzyme (ACE) and Angiotensin type I receptor (AT1R) were involved in PM-induced pathological changes. Western blotting for ACE showed similar results. Moreover, the extent of vascular thickening and the Ang II elevation was most prominent in the Nrf2 gene knockout PM exposure group (KOE). Furthermore, the expression of Nrf2 downstream relevant genes (HO1, Nqo1, Gclc, Gsta4) were significantly enhanced in the wildtype PM exposure group (WTE), while those were remarkably suppressed in the Nrf2 gene knockout groups. The ELISA result of monocyte chemoattractant protein-1 (MCP-1) serum levels in the KOE group was significantly higher in relation to that in the Nrf2 knockout control group (KOC). In summary, PM exposure is associated with thickening of vascular wall, while Nrf2 knockout may further enhance this effect. A potential mechanistic contributor of such effects is the activation of ACE/ANGII/AT1R axis, in which Nrf2 played a regulatory role.

## Introduction

Particulate matter (PM), one of the major hazardous component of air pollution, includes “inhalable coarse particles” with a diameter of 2.5 to 10 μm (PM10) and “fine particles” which are smaller than 2.5 μm in diameter (PM2.5) (Kim et al., 2015). Consistent evidences from both epidemiological and experimental studies have suggested that exposure to PM is related to cardiovascular morbidity and mortality (Li et al., 2017b; Hamatui and Beynon, 2017). Importantly, oxidative stress and endothelial dysfunction have been largely identified as the main alterations involved in the pathogenesis of CVD (Incalza et al., 2018). Previous studies have indicated that PM induced oxidative stress and inflammation in the vascular endothelial cells (Moller et al., 2016), ultimately leading to the endothelial dysfunction and the proliferation of smooth muscle cells (Moyer et al., 2002; Nurkiewicz et al., 2011). In view of the above, further investigation is urgently needed for a better understanding of PM-induced vascular injuries.

Angiotensin II is the main bioactive peptide of the renin-angiotensin system, which is involved in the vascular injuries via inducing vasoconstriction, oxidative injuries and inflammatory reactions, proliferation of vascular smooth muscle cells (VSMCs) and endothelial dysfunction (Li et al., 2017a; Marshall et al., 2017). It is obtained from its precursor, angiotensinogen (AGT), which is firstly converted by renin to produce angiotensin I (Ang I), and then Ang I is converted to Ang II by angiotensin-converting enzyme (ACE) (Paul et al., 2006). The biological/pathological effects of Ang II are mainly mediated by angiotensin II type 1 receptor (AT1R), which belongs to the 7-transmembrane receptor or G protein-coupled receptor (GPCRs) super-family (Balakumar and Jagadeesh, 2014). Therefore, it is well-accepted that dysregulation of circulatory/systemic or local/tissue ACE/Ang II/AT1R axis components can lead to vascular tissue related damages, including effects on vascular tone and proliferation of vascular smooth muscle cells (Daemen et al., 1991; Te Riet et al., 2015; Xu et al., 2017a). Recent reports have provided evidences that PM_2.5_ exposure altered the Ang II and ACE expression (Wang et al., 2016; Zhang et al., 2018). Delfino et al.reported that the expression levels of AGT, ACE and AT1R were significantly increased in endothelial cells of the rat aorta following PM_2.5_ exposure (Delfino et al., 2005). However, under various pathophysiological conditions, the exact source and relevant mechanisms of circulating and locally enhanced renin-angiotensin components have not been established.

It is well recognized that oxidative stress is among the major mechanisms of PM-induced vascular injury, in which the nuclear factor E2-related factor 2 (Nrf2) signaling pathway plays a critical role. Under oxidative or electrophilic stress conditions, ROS promote the breakdown of the Nrf2-Keap1-Cullin3 complex, allowing Nrf2 to heterodimerize with Jun and small musculoaponeurotic fibrosarcoma (sMaf) proteins, which then translocate to the nucleus and binds to the antioxidant response element (ARE) or the electrophile-response element (EpRE) in the promoter region of Nrf2 target genes. As a result, the activated Nrf2 regulates the proteins with antioxidant activities, such as superoxide dismutase (SOD), catalase (CAT), heme-oxygenase 1 (HO-1), glutathione peroxidase 1 (GPx-1) and NAD(P)H: quinone oxidoreductase 1 (Tebay et al., 2015). Recent evidences suggested that PM_2.5_ could exert oxidative injuries on rat aortic endothelial cells, and the expression of Nrf2 protein in vascular endothelial cells of rats was enhanced (Feng et al., 2016; Guan et al., 2019). Nrf2 may be implicated in vascular endothelial injury induced by PM (Xing et al., 2016). Previous study have identified a mechanism underlying Nrf2 activation by oxidative stress that stimulates intrarenal renin-angiotensin system (RAS) gene expression and activation, leading to the development of hypertension (Zhao et al., 2018). However, little information is available as to how Nrf2 affects the expression of renin-angiotensin system (RAS) components.

In this study, we investigated the toxic effects of PM on aorta tissue in C57/B6 mice, which in the individual ventilated cage (IVC)-based real-ambient PM exposure system, focusing on the expression of RAS elements and potential association with Nrf2 gene. The current study adds to the knowledge base of PM-induced vascular injuries and provides scientific evidence for the role of Nrf2 in angiotensin II-associated vascular injury under real-ambient particulate matter exposure in C57/B6mice.

## Methods and Materials

### Animal Grouping and Housing

The Nrf2 gene knockout and wildtype C57/B6 mice (littermates) were obtained from the animal laboratory of School of Public Health, China Medical University, which had been proven to be a successful model for the Nrf2 functional investigation (Itoh et al., 1997). Upon arrival, the mice were adapted for 24 h. And then the wildtype littermate mice were randomly divided into wildtype control (WTC) or wildtype exposure (WTE) groups, while the knockout mice were randomly divided into knockout control (KOC) or knockout exposure (KOE) groups. During the period of experiment, the mice were kept under standard housing conditions (12-h lighting/dark cycle, 22–24°C room temperature, 40–60% room humidity, water and food were provided ad libitum).

General parameters, including the body weight, activity and food intake of the mice, were recorded, and organ indexes were calculated. All procedures used in the current study have been approved by the Institutional Animal Care and Use Committee of Qingdao University and Hebei Medical University in keeping with the National Institutes of Health guidelines.

### IVC Exposure

The IVC exposure method is as described in Li et al. (2019). Briefly, the unique exposure system consists of two separate chambers. One is the air filtered (AF) control chamber, where three high efficiency particulate air filters (HEPA) were placed in to remove all particulate matters. The other one is the PM exposure chamber, where the PM2.5 concentration inhaled is identical with the outdoor atmospheric concentration. Both chambers have identical conditions such as temperature (20–25°C), humidity (40–60%), pressure (15–20 Pa), ventilation frequency (18–20/h), air-flow rate (0.17 m/s), and noise (30–35 dB). Animals were housed in ventilation cages (five per cage) and exposed to unfiltered air or filtered air for 24 h/day and 7 day/week with ad libitum access to food and water. The concentrations of particulate matters in the chambers were detected with an Aerosol Detector DUSTTRAKTM II and analyzed with an Aerodynamic Particle Sizer Spectrometer 3,321 (TSI Incorporated, Shoreview, MN, United States). Meanwhile, the uniformity of PM2.5 distributions was determined by monitoring PM2.5 concentration in ventilation rooms of different racks in the exposure system.

### Sample Collection

Upon desired time points, the animals were anaesthetized with 10 mg/kg bw sodium pentobarbital via intraperitoneal injection and sacrificed. Blood, Heart, lung, liver, spleen, aorta and kidney were collected. Tissues for histological assessments were fixed with 4% paraformaldehyde, and then histologically processed, while other organs were archived in −80°C freezer until further use.

### Blood Routine Examination

The peripheral blood cell number was determined by through routine blood test. Briefly, blood samples were taken from abdominal aorta for blood routine examination. Red blood cell (RBC), hemoglobin (Hb), white blood cell (WBC), white blood cell classification, platelet (PLT) as well as other parameters were counted by automatic blood routine analyzer (HEMAVET 950FS).

### ICP Mass

The blood samples from each 11-weeks group (WTC, WTE, KOC and KOE) were randomly selected and subjected to ICP Mass spectrometry (Agilent 7500CX, CA, United States) for the detection of metals as described in Cui et al. (2020). Briefly, 0.2 ml samples were digested by mixing with 6 ml nitric acid, 1 ml hydrogen peroxide and heated to 200 degrees Celsius for 30 min. The resulting solution was calibrated to 10 ml with ultrapure water and then subjected to ICP-Mass.

The parameters used were high-salt nebulizer, quartz nebulization chamber, quartz glass rods, nebulization temperature: 2 degree Celsius, RF power 1,500 W, Carrier gas 1.25 L/min, Sample depth 7.5 mm, helium 5 ml/min, and Flow rate (sample) 15 ml/min. The metal concentrations in the samples were calculated with the following equation: Concentration (mg/kg) = Acquired reading (ng/ml) × 10/sample mass (g) × 1,000. The limit of detection was 0.005 mg/kg, while the limit of quantification was 0.01 mg/kg. Blank samples and standards were included in each batch of samples. The variation between two independent measurements on the same sample was below 10%.

### Histopathological Assessment

The aortic tissue was fixed in 4% paraformaldehyde for 24 h, and embedded in paraffin. Cross-sections of the aortic tissue were made with a Leica RM2160 microtome at six-micron thickness. Sections were then stained with hematoxylin and eosin (Beyotime, Beijing, China) according to the protocol provided by manufacturer. Pictures of the sections were taken with OlympusBX59, and then ImageJ (NIH, United States) was used to assess the thickness of the vascular wall.

### Immunohistochemistry

The expression levels of ACE and AT1R were evaluated with immunohistochemical staining of the aortic tissue. Sections of aortic tissue were incubated with ACE (1: 1,000) (Beijing Bioss biotechnology co. LTD) or AT1R (1: 1,000) (Proteintech Group, Inc. LTD)antibodies at 37°C for 1h, washed with PBS (pH 7.4) and then incubated with secondary antibody for 20 min. Sections were treated with Biotin-labeled Goat Anti-Rabbit IgG, developed with freshly prepared DAB solution, and counterstained with hematoxylin. Pictures were taken with a microscope (Changfang, Shanghai, China), and quantified with ImageJ software (NIH, United States).

### qRT-PCR

Trizol reagent (Invitrogen, Thermo Fisher Scientific, United States) was used to extract the total RNA from the aortic tissue. Purity and quantification of RNA was determined with Nanodrop One (Thermo Scientific, Waltham, Massachusetts, United States). Total RNA was then reverse-transcribed into cDNA using the primescripttm RT Master Mix kit (Takara Biomedical Technology Co. LTD). And then quantitative RT-PCR was performed with a Power SYBR Green PCR Master Mix kit (Yisheng, Shanghai, China) on a QuantStudioTM 6 FleX Real-Time PCR System (Thermo Fisher Scientific, US). The reaction conditions were as follows: 95°C for 10 min, 40 cycles of 95°C for 10 s, and 60°C for 1 min. The data were analyzed with the 2^−△△Ct^ method. Three independent samples were assessed per group. Primer sequences are reported in Table 1.

**TABLE 1:** Primer sequences used in qRT-PCR.

Name	Forward	Reverse	Gene ID	Association number
PPP2CB	GCT​TTT​ATG​ATG​AGT​GCC​TAC​G	GGT​CCA​GTG​TAT​CTA​TGG​ATG​G	19053	NM_017374.3
AKT1	TGC​ACA​AAC​GAG​GGG​AAT​ATA​T	CGT​TCC​TTG​TAG​CCA​ATA​AAG​G	11651	NM_001165894.1
MAPK1	ATC​TCA​ACA​AAG​TTC​GAG​TTG​C	GTC​TGA​AGC​GCA​GTA​AGA​TTT​T	326413	NM_001038663.1
DDIT3	CTC​CAG​ATT​CCA​GTC​AGA​GTT​C	ACT​CTG​TTT​CCG​TTT​CCT​AGT​T	13198	NM_001290183.1
Tak1	CCC​TTC​AAT​GGA​GGA​AAT​TGT​G	CTC​CAA​GCG​TTT​AAT​AGT​GTC​G	22026	NM_001347342.1
TGFbeta1	CCA​GAT​CCT​GTC​CAA​ACT​AAG​G	CTC​TTT​AGC​ATA​GTA​GTC​CGC​T	21803	NM_011577.2
BMP2	AGT​AGT​TTC​CAG​CAC​CGA​ATT​A	CAC​TAA​CCT​GGT​GTC​CAA​TAG​T	12156	NM_007553.3
Dag1	CTC​CTT​GAA​CCA​GAA​TAG​CGT​C	ATA​ACC​AAG​TTG​GGC​AGA​CAT​A	13138	NM_001276481.1
Abca1	CCT​CAG​AGA​AAA​CAG​AAA​ACC​G	CTT​TGC​TAT​GAT​CTG​CAC​GTA​C	11303	NM_013454.3
AGT	GGT​CTC​TTT​CTA​CCT​TGG​ATC​C	GAC​CTT​GTG​TCC​ATC​TAG​TCG	11606	NM_007428.4
ACE	CTA​CCC​CCA​AGC​ATC​TAT​ACA​G	CCA​CTC​CTG​GTT​ATA​GTT​CTC​C	11421	NM_001281819.2
AT1R	AGC​ATC​TTC​TAC​TCG​AGT​GTT​G	GCG​TCT​GAT​GAT​GAG​TCA​ATT​G	11610	NM_001301281.1
Nqo1	GAA​GAC​ATC​ATT​AAA​CTA​CGC​C	GAG​ATG​ACT​CGG​AAG​GAT​ACT​G	18104	NM_008706.5
Gclc	CTA​TCT​GCC​CAA​TTG​TTA​TGG​C	CCT​CCC​GTG​TTC​TAT​CAT​CTA​C	14629	NM_010295.2
Gsta4	AGT​ACC​CTT​GGT​TGA​AAT​CGA​T	GGT​CCT​TCC​CAT​ACA​AGT​TGT​A	14860	NM_010357.3
HO1	TCC​TTG​TAC​CAT​ATC​TAC​ACG​G	GAG​ACG​CTT​TAC​ATA​GTG​CTG​T	15368	NM_010442.2

Primers were designed and synthesized by Sangon Biotech (Shanghai, China). Store at −20°C. Dissolved concentration is 100 μM.

### ELISAs

Serum Ang II concentrations were determined using a commercially available Ang II ELISA kit (Shanghai Enzyme-linked Biotechnology Co. LTD), while serum monocyte chemoattractant protein-1 (MCP-1) concentrations were measured with a MCP-1 ELISA kit (Abcam biotechnology Co. LTD) in accordance with the manufacturer's instructions.

### Western Blotting

The aortic vessel tissues were lysed in RIPA buffer (Beyotime, Beijing, China) with 1:100 PMSF (Beyotime, Beijing, China) and 1:100 phosphatase inhibitor cocktail (Epizyme, Shanghai, China) added for 30 min, and then centrifuged at 14,000 g, 10 min. Protein concentration was determined using a BCA kit (Beyotime, Beijing, China) following instructions from the manufacturers. 50 μg proteins per sample were electrophoresed in 10% SDS-PAGE. Proteins were then transferred from the gel to polyvinylidene flfluoride (PVDF) membranes and probed with primary antibodies (AT1R antibodies from Proteintech Group, Inc. LTD, dilution ratios were 1:1,000; internal control was GAPDH from Bioss, with a dilution ratio of 1:6,000.) and secondary antibodies (Epizyme goat anti rabbit/mouse IgG at 1:5,000). The bands were visualized with a Fusion Solo S (Vilber Lourmat, Collégien, France) and quantified with ImageJ (NIH, United States) software.

### Statistics Analysis

All the data presented in this paper were collected from at least three independent experiments and the data were expressed as mean ± SD unless otherwise indicated. Statistical analysis was performed with SPSS 24.0. Factorial design ANOVA (two by two) was used to assess differences among groups. All pairwise *p*-values were two-sided and the level of significance was set at *p* < 0.05.

## Results

### Particulate Matter in IVC System and Ambient

The IVC system was constructed to mimic human PM exposure. The concentrations of PM in the IVC system and ambient air during the experiment had been reported in our previous study (Li et al., 2019). The concentration of PM in air filtered (AF) control chambers was undetectable, suggesting that the AF control system was an efficient barrier to remove fine and ultrafine fractions of ambient PM. The diameter of most PM in the exposure chamber was less than 2.5 μm. Although the proportion of fine and ultrafine PM in the exposure chamber was much higher than that in ambient air, the spectrum of the PM number was well correlated with each other when particle sizes were less than 2.5 µm. According to the new standard for air quality assessment, the number of severe contamination days was 12 during the exposure period. The average PM2.5 concentration in the system was about 2.3 times the mean daily limit of 35 µg/m^3^ (Air Quality Guidelines of China). We also conducted a continuous monitoring of outdoor air pollutants, including CO, NO_2_, SO_2_ and O_3_, for 42 days. No significant changes in these outdoor air pollutants were observed during the exposure period. Taken together, this PM exposure system replicated a real-ambient and around-the-clock PM exposure scenario for experimental animals, which resembled the natural state of human exposure.

### General Parameters

The general parameters of 6-weeks exposed animals, including body weight, heart weight, heart index, liver weight, liver index, kidney weight, kidney index, spleen weight, spleen index, lung weight and lung index, were reported in our previous study (Cui et al., 2020). At 6-weeks’ time point, only liver weight (both absolute weight and relative weight) seemed to be elevated significantly. No other statistical differences were observed.

### Blood Routine and ICP-Mass Results

It has been reported in our previous study that the number of white blood cells, lymphocytes and neutrophils under PM exposure were all significantly increased when compared to control group.

After Nrf2 knocked, the number of monocytes was significantly increased in PM exposure group (Jiang et al., 2020). However, the mean value of other parameters, RBC, HCT, MCV, MCH, MCHC, PLT, MPV, HGB, RDW, did not show remarkable changes under PM exposure (*p* > 0.05). For separated data of these blood indexes, please refer to the Supplementary Table 1.

In addition to the hematological assessments, ICP-mass spectrometry was utilized to analyze metal levels in the mice serum. The levels of major metal elements (Na, Mg, Ni, Cu, Al, K, Zn, Se, Ca, Cr, Sr, Ba, Mn, Fe, and Pb) were assessed, and the results are reported in Table 2. Since no significant differences were observed between wildtype and knockout animals receiving same treatment (clean air or PM exposure), samples were pooled to demonstrate the deposition of metals following PM exposure. The results showed that the serum metal contents were not remarkably affected by PM exposure.

**TABLE 2:** The analysis of serum metals contents.

	Control (mg/kg) (n = 10)	Exposure (mg/kg) (n = 10)	*p*
Na	3,269.370 ± 169.823	3,125.942 ± 151.135	0.096
Mg	62.312 ± 13.464	56.160 ± 9.177	0.304
Ni	0.251 ± 0.220	0.185 ± 0.090	0.448
Cu	0.894 ± 0.629	0.714 ± 0.261	0.468
Al	56.878 ± 28.455	36.850 ± 16.646	0.108
K	244.883 ± 50.303	208.130 ± 25.562	0.087
Zn	8.465 ± 4.103	7.660 ± 4.832	0.725
Se	0.601 ± 0.179	0.519 ± 0.138	0.325
Ca	96.102 ± 25.054	98.517 ± 18.934	0.831
Cr	1.417 ± 0.415	1.340 ± 0.553	0.758
Sr	0.768 ± 0.315	0.583 ± 0.251	0.214
Ba	1.467 ± 0.635	1.099 ± 0.476	0.211
Mn	0.627 ± 0.714	0.235 ± 0.063	0.144
Fe	39.490 ± 13.756	38.255 ± 24.599	0.903
Pb	0.209 ± 0.108	0.217 ± 0.178	0.918

### Histopathological Assessments

**FIGURE 1:**
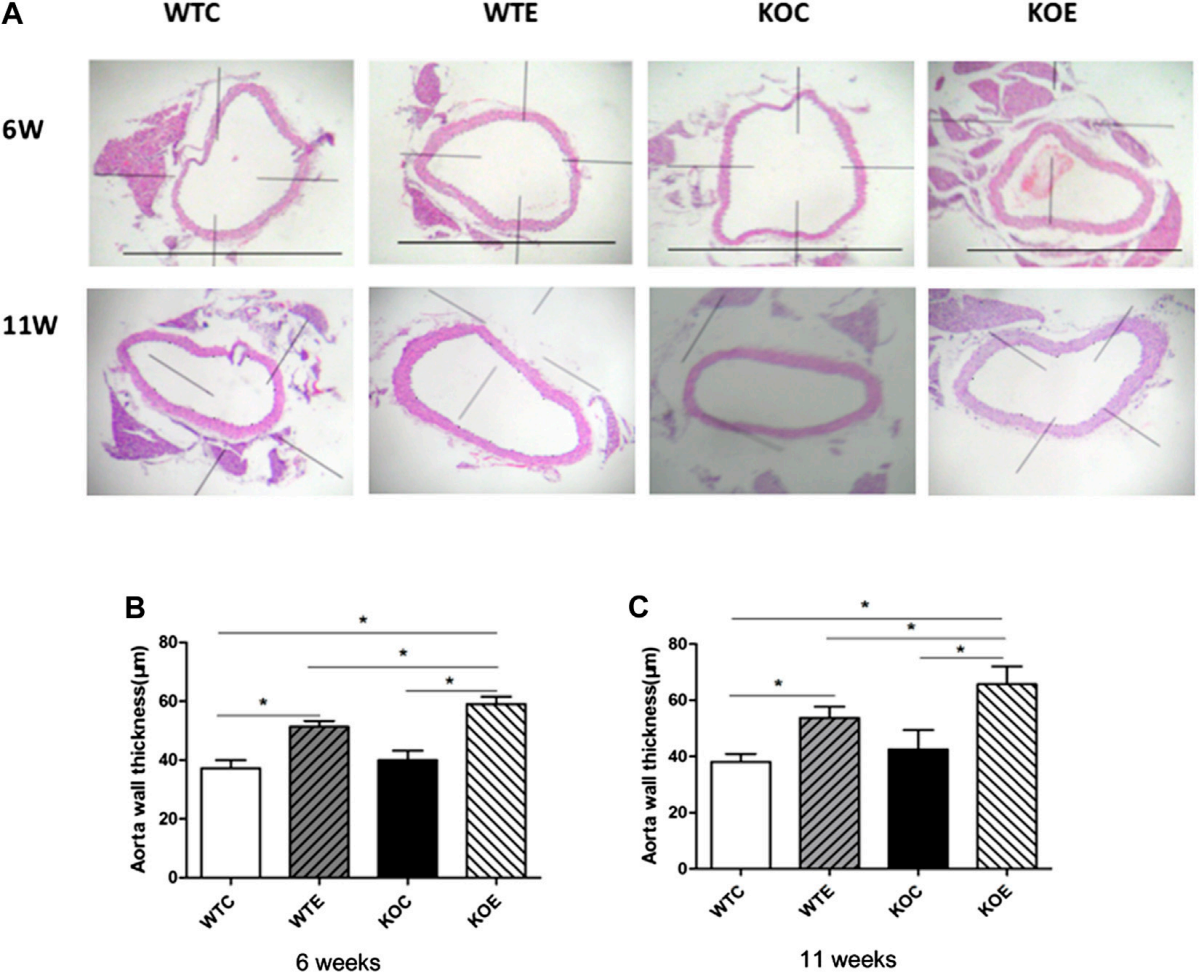
Histopathology results 6–8 weeks C57/B6 mice, with or without Nrf2 knockout were exposed to either filtered air or air containing particulate matter (PM) for six or eleven weeks, and then the vascular tissue samples were fixed in 4% paraformaldehyde for 24 h, and then histologically processed for hematoxylin and eosin staining. The right vascular wall thickness was then measured and analyzed. N = 6 per group. WTC was C57 wild-type control group, WTE was C57 wild-type PM exposure group, KOC was Nrf2-/- group control group, and KOE was Nrf2-/- group PM exposure group. *: statistically different between the two groups (*p* < 0.05). **(A)**: Representative hematoxylin and eosin stained sections for WT-C, WT-E, KO-C, and KO-E groups after 6- or 11-weeks treatment. Scale bars represent 1,000 um. **(B)**: Quantification of the aorta wall thickness following 6-weeks. **(C)**: Quantification of the aorta wall thickness following 11-weeks

After 6-weeks or 11-weeks exposure, histopathological assessment revealed significantly thickening in the aorta wall of the PM exposure group animals relative to those of without PM exposure group animals (Figure 1A). Among PM exposure groups, significant thickened aorta wall can also be observed in Nrf2 knockout group. The quantitative results of aorta wall thickness in each group of mice were reported in Figures 1B,C.

### qRT-PCR Results

**FIGURE 2:**
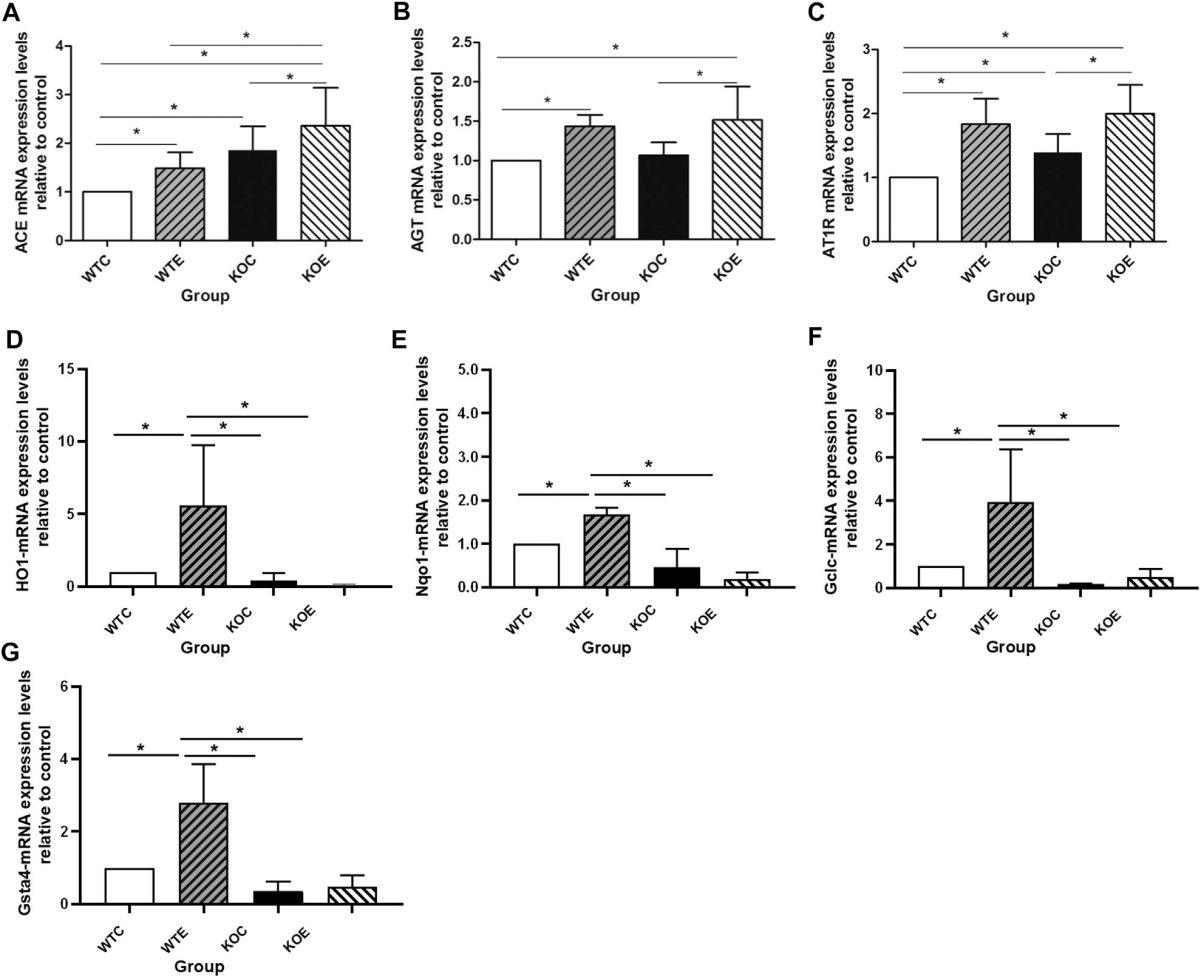
QRT-PCR Results 6–8 weeks C57/B6 mice, with or without Nrf2 knockout were exposed to either filtered air or air containing particulate matter (PM) for six weeks, and then the vascular tissues were collected, snap-frozen in liquid nitrogen. N = 3 per group. WTC was C57 wild-type control group, WTE was C57 wild-type PM exposure group, KOC was Nrf2-/- group control group, and KOE was Nrf2-/- group PM exposure group. *: statistically different between the two groups (*p* < 0.05). **(A)**: Expression of ACE mRNA. **(B)**: Expression of AGT mRNA. **(C)**: Expression of AT1R mRNA. **(D)**: Expression of HO-1 mRNA. **(E)**: Expression of Nqo-1 mRNA. **(F)**: Expression of Gclc mRNA. **(G)**: Expression of Gsta-4 mRNA.

In the current study, we assessed the mRNA expression levels of genes (ABCA1, Dag1 and BMP2) related to the cholesterol, lipid formation and vascular calcification; and mRNA levels of genes (TGFβ, AKT1, MAPK, DDIT3, PRKCB, PPP2CB, ACE, AT1R and AGT) related to vascular smooth muscle proliferation and vascular endothelial injury. Three aorta samples from each group of mice were randomly selected, and subjected to qRT-PCR assay. The expression levels of ACE significantly elevated in WTE, KOC and KOE group relative to those in WTC group, and that in KOE group were also remarkably elevated comparing to those in WTE and KOC groups (*p* < 0.05) (Figure 2A). Moreover, the expression of AGT showed detectable changes between PM exposure group and control group (Figure 2B). In addition to the changes between the exposure and control group, an obvious difference of the expression of AT1R can be observed between KOC group and WTC group (Figure 2C). Another 6 genes related to vascular smooth muscle proliferation and vascular endothelial injury (Table 3) have no significant difference between groups.

**TABLE 3:** Relative expression of gene related to vascular injury *(Mean ± standard deviation).

Genes	Groups			
	WTC	WTE	KOC	KOE
Related to cholesterol, lipid formation and vascular calcification				
ABCA1	1.00 ± 0.00	1.03 ± 0.15	1.14 ± 0.08	1.05 ± 0.17
Dag1	1.00 ± 0.00	1.12 ± 0.03	1.04 ± 0.10	1.11 ± 0.19
BMP2	1.00 ± 0.00	1.01 ± 0.23	1.09 ± 0.09	0.99 ± 0.16
Related to vascular smooth muscle proliferation and vascular endothelial injury				
TGFβ	1.00 ± 0.00	0.92 ± 0.07	0.94 ± 0.11	1.02 ± 0.16
AKT1	1.00 ± 0.00	1.12 ± 0.15	0.97 ± 0.15	1.08 ± 0.29
MAPK	1.00 ± 0.00	0.99 ± 0.12	0.91 ± 0.31	1.04 ± 0.10
DDIT3	1.00 ± 0.00	0.93 ± 0.11	1.13 ± 0.19	1.02 ± 0.22
PRKCB	1.00 ± 0.00	0.94 ± 0.12	1.10 ± 0.31	0.98 ± 0.52
PPP2CB	1.00 ± 0.00	1.06 ± 0.23	0.77 ± 0.13	1.03 ± 0.28

* Values are mean ± SD. WTC was C57 wild-type control group, WTE was C57 wild-type PM exposure group, KOC was Nrf2^-/-^ group control group, and KOE was Nrf2^-/-^group PM exposure group. There is no statistical difference in the expression of the above genes (*p* > 0.05).

qRT-PCR was also performed to confirm the mRNA level of Nrf2 downstream relevant genes (Figures 2D–G) to test and verify the effectiveness of knockout. Among the four genes tested, the expression levels of HO-1, Nqo-1, Gclc and Gsta-4 exhibited similar changes: there is a significant downregulation in the Nrf2 knockout group, while the PM exposure seemed to effectively increase the expression levels comparing with the control group (WTC group and KOC group).

### Immunohistochemistry

**FIGURE 3:**
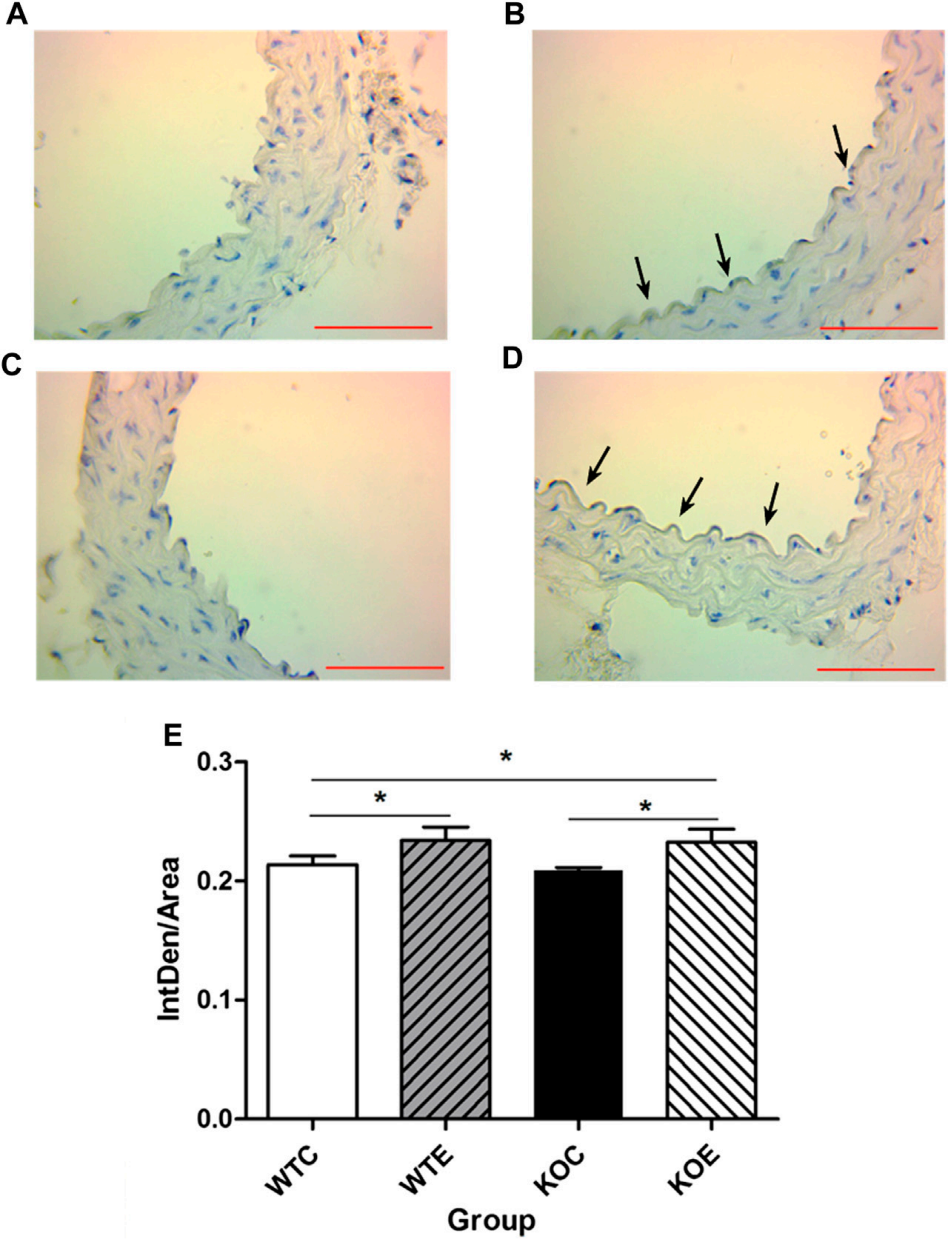
Immunohistochemistry Results of ACE 6–8 weeks C57/B6 mice, with or without Nrf2 knockout were exposed to either filtered air or air containing particulate matter (PM) for six weeks, and then the vascular tissues were collected, embedded in paraffin and subjected to immunohistochemistry for ACE. N = 6 per group. WTC was C57 wild-type control group, WTE was C57 wild-type PM exposure group, KOC was Nrf2-/- group control group, and KOE was Nrf2-/- group PM exposure group. Scale bars are 1,000 μm and magnification is 400×. *: statistically different between the two groups (*p* < 0.05) **(A)**: Immunohistochemistry Results of ACE in WTC group **(B)**: Immunohistochemistry Results of ACE in WTE group **(C)**: Immunohistochemistry Results of ACE in KOC group **(D)**: Immunohistochemistry Results of ACE in KOE group **(E)**: Quantification of the ACE expression.

**FIGURE 4:**
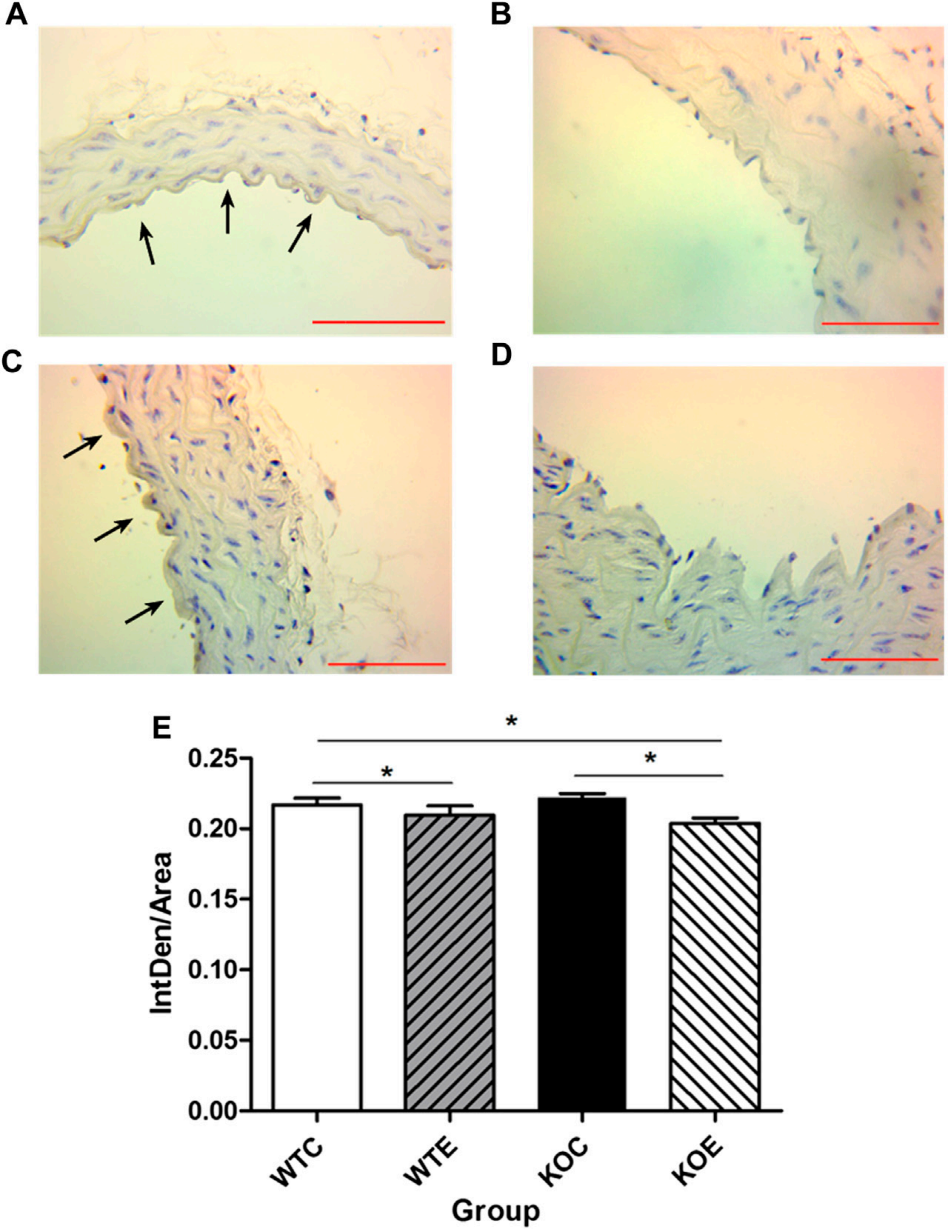
Immunohistochemistry Results of AT1R 6–8 weeks C57/B6 mice, with or without Nrf2 knockout were exposed to either filtered air or air containing particulate matter (PM) for six weeks, and then the vascular tissues were collected,embedded in paraffin and subjected to immunohistochemistry for AT1R. N = 6 per group. WTC was C57 wild-type control group, WTE was C57 wild-type PM exposure group, KOC was Nrf2-/- group control group, and KOE was Nrf2-/- group PM exposure group. Scale bars are 1,000 μm, and magnification is ×400. *: statistically different between the two groups (*p* < 0.05). **(A)**: Immunohistochemistry Results of AT1R in WTC group. **(B)**: Immunohistochemistry Results of AT1R in WTE group. **(C)**: Immunohistochemistry Results of AT1R in KOC group. **(D)**: Immunohistochemistry Results of AT1R in KOE group. **(E)**: Quantification of the AT1R expression.

To further determine the potential sources responsible for circulating augmented Ang II and the mechanism of ACE/Ang II/AT1R axis in the vascular injury induced by PM, the levels of AT1R and ACE, the major components of RAS, were assessed with immunohistochemistry. The results revealed that the expression levels of ACE on intima in the exposed groups were higher than those in the unexposed groups (Figure 3), while the expression levels of AT1R in the exposed groups were lower than those in the unexposed groups on intima (Figure 4).

### Western Blotting Results

**FIGURE 5:**
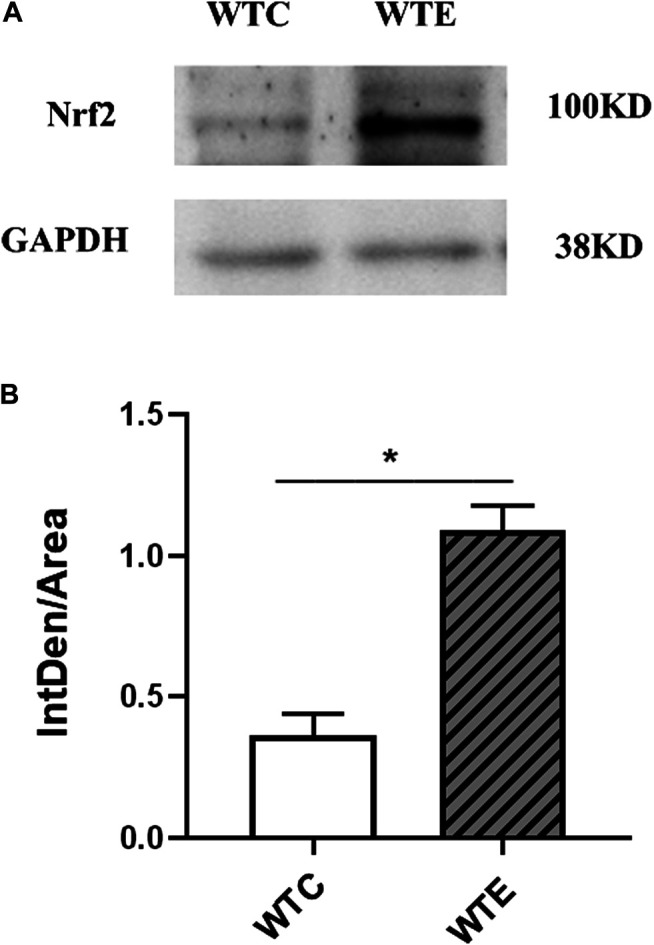
Western Blotting Results of Nrf2 6–8 weeks C57/B6 mice, with or without Nrf2 knockout were exposed to either filtered air or air containing particulate matter (PM) for six weeks, and then the vascular tissues were collected, embedded in paraffin, extracted and subjected to western blotting for Nrf2. N = 4 per group. *: statistically different between the two groups (*p* < 0.05). **(A)**: The expression level of Nrf2 in vascular tissue. **(B)**: Quantification of the Nrf2 expression.

**FIGURE 6:**
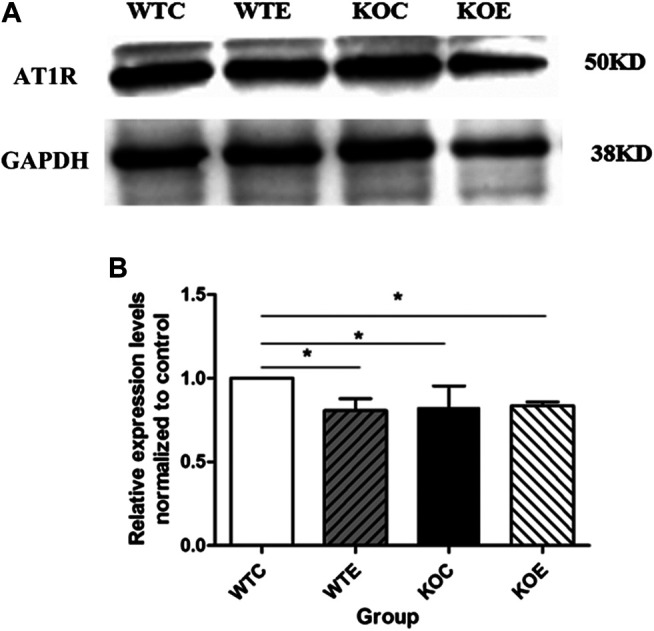
Western Blotting Results of AT1R 6–8 weeks C57/B6 mice, with or without Nrf2 knockout were exposed to either filtered air or air containing particulate matter (PM) for six weeks, and then the vascular tissues were collected, embedded in paraffin, extracted and subjected to western blotting for AT1R. N = 4 per group. *: statistically different between the two groups (*p* < 0.05). **(A)**: The expression level of AT1R in vascular tissue. **(B)**: Quantification of the AT1R expression.

The expressions of Nrf2 were determined by Western blotting. The results showed that expression level of Nrf2 gene in wild type mice was remarkably increased under PM exposure when compared to control group (Figure 5). To further verify the changes of AT1R at the translational and post-translational levels, western blotting was performed on the vascular tissue samples of each group. The results showed that the AT1R expression levels were significantly decreased following PM exposures and Nrf2 knockout (Figure 6).

### ELISA Assays

**FIGURE 7:**
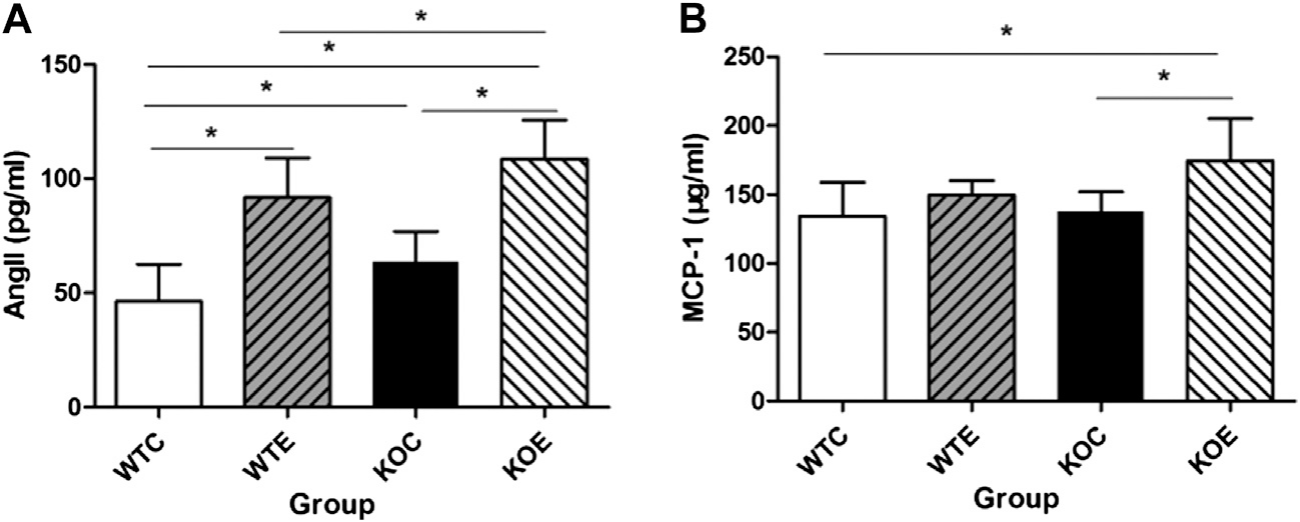
ELISA Results of Ang II and MCP-1 6–8 weeks C57/B6 mice, with or without Nrf2 knockout were exposed to either filtered air or air containing particulate matter (PM) for six weeks, and then the blood was subjected to ELISA assayfor Ang II and MCP-1. *: statistically different between the two groups (*p* < 0.05). N = 10 per group. **(A)**: The serum concentration of Ang II. **(B)**: The serum concentration of MCP-1.

To determine the role of Ang II in PM-induced vascular damage, ELISA was utilized to determine the serum levels of Ang II among the groups. As shown in Figure 7A, exposure to PM led to significant elevation of plasma concentration of Ang II and this change was more pronounced in KOE group. The serum level of MCP-1 was also measured with ELISA (Figure 7B). The data revealed that the significant increase of KOE group existed relative to KOC and WTC group.

## Discussion

Previous studies have demonstrated that PM exposure is associated with elevated concentration of serum Ang II (Xu et al., 2017b), and the latter played a crucial role in the pathophysiology of cardiovascular disorders by inducing vasoconstriction effect (Zhang et al., 2019). Besides, a number of related researches have proposed that the oxidative stress and its direct consequence of the generation of inflammation were key mechanism of the vascular injury induced by PM exposure (Nel, 2005). And the induction of Nrf2-induced phase II enzyme expression together with the Nrf2 up-regulation act as sensitive markers for oxidative stress, suggesting the triggering of Nrf2-driven antioxidant response (Araujo et al., 2008). Our current study has focused on the vascular effects and the mechanism of ACE/Ang II/AT1R axis in PM-induced vascular injury of Nrf2-knockout animal. Better relevancy was achieved by employing an IVC system thus subjected experimental animals to real-world ambient PM in Shijiazhuang, China. Meanwhile, the Nrf2-knockout mice were used to determine whether the major antioxidant gene Nrf2 plays a role in PM-mediated vascular injury.

It is generally accepted that exposure to PM can change the vasoconstriction/vasodilation state of blood vessels, leading to vascular endothelial injury and/or vascular dysfunction (Schneider et al., 2008; Krishnan et al., 2012), as well as changes in blood pressure, blood lipid and thickness of vascular wall (Martinez-Lemus et al., 2009; Chappell et al., 2016; Tuder, 2017). Similarly, in the current study, the histological assessments revealed thickening of vascular upon PM exposure. It has been reported that the thickening of the vascular wall is related to the increase of intravascular cholesterol and lipid deposition, calcification of vascular wall, proliferation of vascular smooth muscle and vascular endothelial injury (Rajendran et al., 2013; Leopold, 2015). However, the mRNA results indicated no significant change in the genes related to cholesterol lipid production and vascular calcification (ABCA1, Dag1, BMP2). Further studies are needed regarding the exact molecular mechanism for vascular thickening induced by PM exposure.

PM is involved in the vascular injury via inflammation reaction (Pope et al., 2016). When the body is challenged by external stimulants, neutrophils enter the surrounding tissue through the capillary wall under the action of chemokines, concentrate in large numbers in the lesion, and carry out active phagocytosis and secretory activities (El-Benna et al., 2016). Similarly, in our previous study, elevated neutrophils counting indicated that PM served as an external stimulant to the body, inducing inflammatory responses (Jiang et al., 2020). Hemolysis is the premature destruction of red blood cells (RBCs) membranes and it can be induced by the mineral components adsorbed on the particles (Mesdaghinia et al., 2019). In the current study, no significant changes were observed in the relevant red blood cell index, which indicates that PM did not cause the destruction of RBCs. According to earlier studies, Nrf2 played a pivotal role in inflammation, in which it can contribute to the anti-inflammatory process by orchestrating the recruitment of inflammatory cells and regulating gene expression through the antioxidant response element (ARE) (Ahmed et al., 2017), while its signaling pathway is under to be further investigated.

Metals elements are important components in air pollutants, in which it has a remarkable bioaccumulation effect (El-Khatib et al., 2020). In the current study, we investigated the potential bioaccumulation of metal elements by ICP-MS. The results showed that the contents of the metal (Na, Mg, Ni, Cu, Al, K, Zn, Se, Ca, Cr, Sr, Ba, Mn, Fe, and Pb) in the serum did not show detectable changes in the mice of PM exposed for 11-weeks. However, we have identified from our previous report that the levels of Na, K, Se, and Fe in the cardiac tissue of mice in the PM exposure group increased significantly (Cui et al., 2020). The accumulation of different metal elements in the body is organ-specific (Siraj et al., 2016). Some elements can be excreted from the body with metabolism, and some of them can be accumulated in fat, lung, liver, etc., so that after a period of PM exposure, the free metal elements in the circulation decreased, and excessive metal accumulated in the target organs (Tchounwou et al., 2012). The finding in our study suggested that the metal elements in the PM might have translocated into the target organs and caused toxicity injuries in them (Jaishankar et al., 2014) without accumulating in the serum.

Previous reports have demonstrated that PM2.5 exposure resulted in abnormal elevation of circulating/systemic or local/tissue AGT/ACEI/AT1R axis components and elevated plasma concentration of Ang II to induce oxidative stress in blood vessels (Daemen et al., 1991; Gwathmey et al., 2010; Xu et al., 2017a). Since the ACE/Ang II/AT1R axis is a cascade reaction, in which any unbalanced expression of any component can lead to the changes in the expression of related factors, thus leading to the vascular injury (Dantas and Sandberg, 2005; Montezano et al., 2014; Feng et al., 2020). The data from the current study demonstrated that PM2.5 exposure resulted in elevation of circulating Ang II levels, suggesting that AngII has contributed to the thickening of the vascular wall. Besides, the upregulation of AGT and ACE in vascular tissue might provide an explanation for the parallel increase in formation of serum Ang II after PM exposure. As one of the crucial components in the RAS, ACE is involved in cell proliferation and remodeling (Han and Ge, 2016). Additionally, it had been reported that activator protein-1 (AP-1)-associated AT1R upregulation contributed to chemokines synthesis, which subsequently recruited monocytes and thus induced a proinflammatory response in the vascular endothelium (Xu et al., 2019). However, in the current research, remarkably decreased AT1R expression levels were observed in PM exposed mice, while the expression level of AT1R mRNA ascended. The inconsistency between AT1R mRNA and protein levels may be related to the transcription and translation process, which can affected by the release of cytokine, such as TNFα (Gurantz et al., 1999) and IL-1 α (Sasamura et al., 1997). The mechanism that affects the transcription and translation process of AT1R gene remains to be further investigated.

At present, the widely recognized mechanism of PM-induced injury is oxidative stress. Among the key factors regulating antioxidant activities, nuclear factor E2-related factor 2 (Nrf2) is a major one, which appeared to be involved in oxidative stress as a protective factor (Guan et al., 2019). Specifically, there was research reported that PM2.5 enhanced the generation of basal ROS and the levels of lipid peroxidation in Nrf2-silenced cells, demonstrating the significance of Nrf2 in maintaining redox balance (Pardo et al., 2019). Moreover, our previous study reported that significantly elevated malondialdehyde levels were observed in two KO groups relative to the corresponding wildtype controls (Cui et al., 2020). In the current study, similar changes were observed: pathological observation indicated more obvious vascular walls thickening in KOE group relative to the other three groups, as was the expression level of ACE gene and the concentration of serum Ang II as well as MCP-1, which participates in the migration and proliferation of vascular smooth muscle cells and the occurrence or development of intimal hyperplasia from different aspects (Huang et al., 2015). And the qRT-PCR results of Nrf2 downstream relevant genes indicated Nrf2 knockout effectively. Meanwhile, the western blotting results of Nrf2 also showed that the PM exposure in wild type mice could induce the Nrf2 up-regulation, which was consistent with the previous report by other group (Guan et al., 2019). Accordingly, the results from Mann et al. (2007) have reviewed the roles of Nrf2 in vascular homeostasis and the defense of endothelial and smooth muscle cells against sustained oxidative stress (Mann et al., 2007). Corresponding researches have indicated that the protective effects of Nrf2 included decreased VSMCs migration, proliferation, calcification and vascular remodeling (Kim et al., 2009; Ashino et al., 2013; Aghagolzadeh et al., 2017). And the result from Canella et al. (2018) has demonstrated that Nrf2 activation prevented lung cells from tissue damage induced by oxidative stress through modulating membrane currents by means of antioxidant response. Furthermore, Yang et al. showed that angiotensin II-induced apoptosis and oxidative stress in vascular endothelial cells might be protected by the activation of Nrf2/ARE signaling (Yang et al., 2019). Taken these facts together, it is suggested that Nrf2 exhibited protective functions on the PM-induced vascular injury through ACE/Ang II/AT1R axis, while its in-depth mechanism needs to be further explored.

## Conclusion

In summary, the data of the current study demonstrated that PM exposure could induce aortic wall thickening in mice, and the potential mechanism may be associated with ACE/AngII/AT1R axis. Besides, Nrf2 gene plays a regulatory role in vascular injury in PM-induced vascular injury through ACE/AngII/AT1R axis, whose mechanism remains to be further clarified.

## Data Availability

The raw data supporting the conclusions of this article will be made available by the authors, without undue reservation.
