# Supplementary material for: The Role of Nrf2 in the PM-Induced Vascular Injury Under Real Ambient Particulate Matter Exposure in C57/B6 Mice
Source: Front Pharmacol. 2021 Feb 26;12:618023. doi: 10.3389/fphar.2021.618023 (PMC7952307; doi:10.3389/fphar.2021.618023)
Supplement: Supplementary file 1 [file table1.docx]

*Supplementary Materials*

Supplementary Table

Table 1 Results of Blood routine test results in mice

| **Group**  **Project** | **WT-C** | **WT-E** | **KO-C** | **KO-E** | **P** |
| --- | --- | --- | --- | --- | --- |
| **RBC（×1012/L）** | 7.352±1.418 | 7.763±0.925 | 6.670±1.369 | 7.717±1.415 | 0.491 |
| **HCT（%）** | 40.100±8.122 | 40.700±4.451 | 32.300±7.982 | 42.100±7.223 | 0.140 |
| **MCV（fl）** | 54.300±2.040 | 52.500±2.440 | 54.800±6.058 | 54.500±1.572 | 0.109 |
| **MCH（pg）** | 12.440±0.833 | 12.650±0.553 | 12.460±1.85 | 12.830±0.58 | 0.113 |
| **MCHC（g/L）** | 229±15.030 | 241±11.490 | 235±34.330 | 236±9.020 | 0.108 |
| **PLT（×109/L）** | 225±63.630 | 319±99.760 | 336±40.000 | 242±116.000 | 0.058 |
| **MPV（fl）** | 5.115±0.552 | 4.687±0.550 | 5.311±0.448 | 4.973±0.764 | 0.080 |
| **HGB（g/L）** | 95.830±14.890 | 98.270±13.520 | 87.710±26.570 | 99.550±20.760 | 0.144 |
| **RDW（fl）** | 16.100±0.660 | 16.390±0.650 | 17.080±1.200 | 16.550±1.000 | 0.101 |
